# DNA Methylation, Aging, and Cancer Risk: A Mini-Review

**DOI:** 10.3389/fbinf.2022.847629

**Published:** 2022-06-02

**Authors:** Larry Chen, Patricia A. Ganz, Mary E. Sehl

**Affiliations:** ^1^ Computational and Systems Biology Program, University of California, Los Angeles, Los Angeles, CA, United States; ^2^ Division of Hematology-Oncology, Department of Medicine, UCLA David Geffen School of Medicine, Los Angeles, CA, United States; ^3^ Department of Health Policy and Management, Fielding School of Public Health, University of California, Los Angeles, Los Angeles, CA, United States; ^4^ Department of Computational Medicine, UCLA David Geffen School of Medicine, Los Angeles, CA, United States

**Keywords:** aging, epigenetic clocks, DNA methylation, carcinogenesis, cancer risk

## Abstract

Accumulation of somatic mutations and genomic instability are hallmarks of both aging and cancer. Epigenetic alterations occur across cell types and tissues with advancing age. DNA methylation-based estimates of biologic age can predict important age-related outcomes, including risk of frailty and mortality, and most recently have been shown to be associated with risk of developing cancer. In this mini-review, we examine pathways known to exhibit altered methylation in aging tissues, pre-malignant lesions, and tumors and review methodologies of epigenetic clocks that reliably predict cancer risk, including those derived from methylation studies of peripheral blood, as well as those methylation levels from within the tissues at high risk of cancer.

## Introduction

Cancer incidence increases exponentially with advancing age, beginning at the midpoint of the lifespan in most mammalian species (Campisi and Yaswen, 20092009). Somatic mutations accumulate within cells with chronic cell cycling (Moskalev et al., 2013), leading to genomic instability, a hallmark of both aging and cancer (Hanahan and Weinberg, 2011; López-Otín et al., 2013). Over the past decade, epigenetic alterations that occur with advancing age across cell types and tissues have been identified (Teschendorff et al., 2010; Horvath, 2013), and methylation markers at select sites have been shown to reliably predict chronologic age (Teschendorff et al., 2010; Bocklandt et al., 2011; Hannum et al., 2013; Horvath, 2013). Epigenetic clocks have been further shown to predict age-related diseases and outcomes, including frailty and mortality, suggesting that they are reliable markers of biologic aging (Chen et al., 2016; Levine et al., 2018; Lu et al., 2019a). Importantly, methylation age has recently also been associated with cancer risk (Levine et al., 2015; Lu et al., 2019a; Yu et al., 2020). In this article, we will review studies of global methylation alterations that occur with advancing age and cancer risk, compare the development and features of first- and second-generation epigenetic clocks, as well as the epigenetic pacemaker clock and other methods, and illustrate their ability to predict risk of incident cancer.

## Pathways With Aberrant Methylation in Malignant Tissues

DNA methylation is thought to play an important role in the etiology of complex traits, including cancer (Esteller, 2008; Petronis, 2010). The importance of DNA methylation in carcinogenesis was recognized with the discovery of numerous hypermethylated promoters of tumor suppressor genes in tumor samples, as well as findings confirming the role of DNA methylation in facilitating DNA damage, e.g., in the silencing of mismatch repair genes (Jones and Laird, 1999). Hundreds of genes, including key tumor suppressor genes, are hypermethylated at promoter CpG islands, and are either transcriptionally silenced or blocked from normal induction, in nearly every patient’s cancer compared with normal cell counterparts (Vaissière et al., 2009; Baylin and Jones, 2016; Xie et al., 2018). The similarity in epigenetic alterations that occur during tumorigenesis and senescence raises the question of whether programmatic changes that occur during senescence play a role in carcinogenesis. Though the promoter hypermethylation events in malignant transformation appear to arise independently of cellular senescence (Xie et al., 2018), further exploration is needed to identify a relationship between cancer risk and epigenetic events occurring in development and aging.

## Age-Dependent Hypermethylation of Polycomb Group Target Proteins

Polycomb group proteins repress genes required for stem cell differentiation, and targets of polycomb group proteins (PCGTs) are repressed in human embryonic and adult stem cells through reversible chromatin modifications (Lee et al., 2006). PCGTs are 12-fold more likely to be methylated in cancer tissues than non-PCGTs, suggesting a mechanism of carcinogenesis where cells are locked in an un-differentiated state of self-renewal and predisposed to malignant transformation. In an analysis of whole blood samples from 261 postmenopausal women, Teschendorff demonstrated that PCGT CpGs are hypermethylated with advancing age, and this methylation signature was validated in seven independent data sets encompassing 900 samples, from multiple cell types and tissues including blood, ovarian cancer, cervix, and marrow mesenchymal stem and stromal cells (Teschendorff et al., 2010).

## Variability in Age-Related Methylation Patterns in Premalignant Lesions

Methylation markers drift differentially with age between normal and premalignant tissues. In pre-malignant dysplastic tissues, age-PCGT CpGs were more highly methylated than in normal samples, suggesting that age may contribute to carcinogenesis by irreversibly silencing genes that are suppressed in stem cells (Teschendorff et al., 2010). Importantly, in dysplastic tissues, differential variability in methylation identifies cancer risk markers more reliably than differences in mean methylation (Teschendorff and Widschwendter, 2012). Differentially variable features identified in precursor non-invasive lesions exhibit significantly increased enrichment for developmental genes compared with differentially methylated sites (Teschendorff and Widschwendter, 2012). In studies of normal and pre-malignant esophageal tissues, differential methylomic drift occurs in Barrett’s esophagus (BE) relative to normal squamous tissue (Curtius et al., 2016). Using a Bayesian model incorporating longitudinal methylomic drift rates, patient age, and methylation data from BE and normal squamous tissue, Curtius et al. have developed a molecular clock to reliably estimate patient-specific BE onset times, providing information about how long an individual has lived with the precursor lesion (Curtius et al., 2016).

## DNA Methylation-Based Estimates of Age

Table 1 summarizes 16 epigenetic clocks that have been developed over the past decade. These clocks reliably estimate chronologic age based on methylation levels at select CpGs (Di Lena et al., 2021). In an early work analyzing methylation patterns in saliva associated with advancing age, lasso penalized regression was used to screen for the top predictors of age, and a leave-one-out regression analysis was used to form an accurate epigenetic predictor of age (Bocklandt et al., 2011). Subsequently, Horvath developed a multi-tissue epigenetic clock across 51 healthy tissues and cell types, that reliably estimated methylation age across cell types and tissues (Horvath, 2013). This epigenetic clock was found to be close to zero for embryonic and induced pluripotent stem cells, applied across species to chimpanzees (Horvath, 2013), and importantly was later found to be accelerated in disease states (Horvath et al., 2014; Rickabaugh et al., 2015) and predictive of frailty and mortality (Chen et al., 2016). This clock utilizes elastic net regression on a transformed continuous, monotonically increasing function of age to select a set of CpGs whose weighted average reliably predict age across a wide spectrum of tissues and cell types. Elastic net regression linearly combines the *l*_1_ (lasso) and *l*_2_ (ridge) penalty terms. While the lasso tends to select only one variable from a group of highly correlated variables, the quadratic expression elevates the loss function toward being convex, allowing a larger number of variables to be included when there is a higher correlation of variables and higher grouping effect. To avoid low efficiency in predictability and high bias from subjecting coefficients to two types of shrinkages, coefficients are rescaled by multiplying them by (1 + *l*_2_). The Hannum clock similarly uses elastic net regression to estimate chronologic age from methylation levels at 71 CpGs in peripheral blood (Hannum et al., 2013). Blood-based epigenetic age measures Intrinsic and Extrinsic epigenetic age incorporate information on cell composition (imputed from methylation data at additional sites) to estimate age. While Intrinsic epigenetic age is independent of changes in cell distribution that occur with advancing age, Extrinsic epigenetic age is positively and negatively correlated with estimated proportions of naïve and senescent cytotoxic T lymphocytes. More recently, second generation clocks have been developed, including Phenotypic age (Levine et al., 2018) and Grim age (Lu et al., 2019a) which are both more closely associated with lifespan, and utilize a two-step process to estimate biologic age. In the development of the Phenotypic age clock, first 1) Cox penalized regression is used to identify a set of biomarkers that best predict aging-related mortality, and next 2) a mortality score based on the regression coefficients from step 1 is converted into units of years and the resultant phenotypic age estimate is regressed on DNA methylation using an elastic net regression analysis. Grim age clock also involves a two-step process in which 1) methylation data is used to estimate smoking pack-years and levels of plasma proteins known to be associated with morbidity or mortality, and 2) time-to-death is regressed on these DNA methylation-based surrogate biomarkers, resulting in a mortality risk estimate that is transformed into units of years. The Skin and Blood clock is a robust estimator of methylation age in fibroblasts, keratinocytes, buccal cells, endothelial cells, lymphoblastoid cells, skin, blood, and saliva, and was developed when it was found that other clocks performed poorly in human fibroblasts and other skin cells (Horvath et al., 2018). A DNA methylation-based estimate of telomere length, DNAmTL, is a measure of cell replicative history, and outperforms measured leukocyte telomere length in predicting time to death and age-related pathologies (Lu et al., 2019b). Stubbs et al. developed an accurate multi-tissue age estimator in mice, with CpGs from pathways involved in morphogenesis and development (Stubbs et al., 2017). Additional clocks have been developed to more accurately predict age in human cortical tissue (Shireby et al., 2020), skeletal muscle (Voisin et al., 2020), and pediatric buccal epithelial (McEwen et al., 2020) tissues. Finally, epigenetic clocks have been developed to predict gestation age using methylation levels of cells from umbilical cord and placental tissues (Bohlin et al., 2016; Knight et al., 2016; Mayne et al., 2017; Lee et al., 2019).

**TABLE 1:** Features of epigenetic clocks.

Clock	Methods	# CpGs	Pathways	Special Features	Ref
Pan-tissue	Elastic net	353	Survival, apoptosis, self renewal	Reliable across cell types and tissues	5
Hannum	Elastic net	71	Cell cycle regulation, DNA repair, iron homeostasis	Widely used in epidemiologic studies	8
Phenotypic	Cox penalized regression + elastic net	513	JAK-STAT cascade, tumor necrosis, NFkB, lipopolysaccharide	Strong predictor of healthspan and lifespan	11
Grim	Cox penalized regression + elastic net	1,030	MHC II, cytokine signaling, protein sumoylation	Strongest predictor of mortality	10
EpiTOC	Informed selection	385	Stem cell renewal and differentiation	Estimated rate of stem cell division	37
Skin and Blood	Elastic net	391	Survival, apoptosis, selfrenewal, cell cycle regulation, DNA repair	Most accurate predictor of age in skin and fibroblasts	25
Epigenetic pacemaker	Conditional expectation maximization	varies	variable	Accounts for nonlinearities in aging rates	27
DNAmTL	Elastic net	140	Cadherin, cell signaling	Marker of cell replicative history	26
Mouse clock	Elastic net	329	Development, morphogenesis	Multi-tissue predictor in mice	28
Human cortex clock	Elastic net	347	None reported	Accurate in human cerebral cortex	29
Skeletal muscle clock	Elastic net	200	None reported	Accurate in human skeletal muscle	30
PedBE clock	Elastic net	94	None reported	Accurate in buccal cells of children	31
Gestational age clocks	Elastic net, lasso	148, 62, 58, 5,474	Cell aging and senescence	Umbilical cord blood and placenta at birth; predictive of gestational age	32–35

Despite high accuracy, epigenetic clocks do not permit characterization of the non-linear epigenetic aging patterns that occur across the entire lifespan. Recently, a new method was developed to model epigenetic changes with age while accounting for the nonlinearities of this relationship that occur with advancing age (Snir et al., 2019). This integrated framework, based on evolutionary models, addresses the acceleration and deceleration of epigenetic changes that occur over time, and has been applied to methylation data from broad age ranges and multiple tissue types. The Epigenetic Pacemaker (EPM) employs a fast conditional expectation maximization algorithm to model epigenetic states associated with a phenotype of interest, such as aging and type 2 diabetes mellitus. In this algorithm, each methylation site is assigned an independent rate of change and starting methylation value, while each individual is assigned an epigenetic state. Given i methylation sites and j individuals, a single methylation site can be described as:

$$\hat{m_{\mathrm{ij}}}\;=\;m_{i}^{0}+\;r_{i}s_{j}+\in_{\mathrm{ij}}$$

Where $\hat{m_{\mathrm{ij}}}$ is the observed methylation value, $m_{i}^{0}$ is the initial methylation value, $r_{i}$ is the rate of change, $s_{j}$ is the epigenetic state, and $\in_{\mathrm{ij}}$ is a normally distributed error term. The goal of the EPM is to find the optimal values of the initial methylation value, rate of change, and epigenetic state to minimize the error between the predicted and observed methylation values across a system of methylation sites. The epigenetic state is then updated through each iteration of the EPM to minimize the error across the observed epigenetic landscape. Because the epigenetic state is updated while fitting the EPM, the assumption of linearity between the methylation values and the phenotypic trait of interest is relaxed. In addition to examining age as an outcome of interest, these models can be employed to study additional phenotypes of interest, including risk of cancer.

## Epigenetic Clocks Predicting Risk of Cancer

Older tissues are at greater risk of malignant transformation because of acquired mutations that occur in the setting of prolonged epithelial proliferation. Several recent studies have demonstrated that accelerated aging in peripheral blood predicts subsequent development of cancer (Levine et al., 2015; Kresovich et al., 2019a; Zheng et al., 2016; Perna et al., 2016; Durso et al., 2017; Kresovich et al., 2019b). Table 2 summarizes epigenetic clocks that have demonstrated association with cancer risk. Pan-tissue clock acceleration in peripheral blood is associated with later development of lung cancer (Levine et al., 2015), breast cancer (Durso et al., 2017), and male colon cancer (Durso et al., 2017). Grim age, a strong predictor of mortality, is associated with time to any cancer (Lu et al., 2019a). Intrinsic epigenetic age acceleration in peripheral blood is associated with risk of post-menopausal breast cancer, with epigenetic acceleration detected up to 10 years prior to cancer diagnosis (Ambatipudi et al., 2017). In a large study examining methylation in 2,764 cancer free women in the Sister Study, 1,566 of whom subsequently developed breast cancer after an average of 6 years, acceleration of Pan-tissue age, Hannum age, and Phenotypic age each predicted risk of subsequent breast cancer (Kresovich et al., 2019b). In this study, Grim age was associated with invasive breast cancer in post-menopausal women (Kresovich et al., 2019a). Using data from seven nested case-control studies of peripheral blood DNA methylation and colorectal, gastric, kidney, lung, prostate, and urothelial cancer, and B cell lymphoma from the Melbourne Collaborative Cohort Study, epigenetic aging was associated with both risk of cancer and increased risk of death after cancer diagnosis (Dugué et al., 2018). A five-year increase in age acceleration was associated with a 4–9% increase in risk of cancer, and a 2–5% increased risk of death following cancer diagnosis (Dugué et al., 2018).

**TABLE 2:** Epigenetic clocks predictive of cancer risk.

Epigenetic clock	Tumor type	Ref
Grim age (blood)	Time to cancer (all), invasive breast cancer in post-menopausal women	10, 28
Intrinsic age (blood)	Breast cancer risk	33
Pan-tissue age (blood)	Lung, breast cancer risk, other cancers	12, 32, 34
Hannum age (blood)	Breast cancer risk	32
Phenotypic age (blood)	Breast cancer risk	32
Pan-tissue (colon cancers)	Cancer subtype, prognosis	35
Pan-tissue (breast tumors)	More aggressive breast cancers in younger women	36
EpiTOC (precancerous lesions and cancer)	Accelerated in precancerous lesions and cancer	37
MiAge calculator (13 cancer types and adjacent normal tissues)	Accelerated mitotic age in tumor tissues is associated with worse survival	47

In addition to the associations found between cancer risk and epigenetic aging in peripheral blood, several studies have examined age-related epigenetic changes in tissues that subsequently develop cancer. Epigenetic aging is associated with cancer risk (in at-risk tissues) and prognosis (in cancerous tissues). For example, Pan-tissue age acceleration in colon cancer samples has been linked with colon cancer molecular subtypes and improved prognosis prediction because it is linked with overall survival (Zheng et al., 2019). In breast tumor samples, methylation studies within the breast of very young women with more aggressive breast cancer exhibit accelerated DNA methylation age compared with breast cancer in older counterparts, suggesting a role of accelerated epigenetic aging in breast cancer risk (Oltra et al., 2019). In addition, methylation-based markers of cell replication have been associated with cancer risk, including the epigenetic mitotic clock (EpiTOC) which approximates the rate of stem cell division in normal tissues by focusing on promoter CpG sites that localize to PCGT genes, and has been shown to be accelerated in precancerous lesions and cancer (Yang et al., 2016). Furthermore, Youn et al. demonstrated that quantitative estimates of mitotic age (total number of cell divisions) of a tissue, derived using the stochastic replication errors accumulated during cell divisions predict shorter disease associated survival in thirteen cancer types studied (Youn and Wang, 2018). In healthy breast tissue, methylation of tumor suppressor genes APC (Lewis et al., 2005; Euhus et al., 2008) and RASSF1 (Lewis et al., 2005) is associated with breast cancer risk as measured by the Gail model risk score. In a recent study comparing disease-free breast tissue cores from women at high versus average risk for breast cancer using the Tyrer-Cuzick model, 1698 DNA methylation aberrations were identified in high-risk breast tissues, from pathways involving cell adhesion, ErbB, and protein kinase A signaling (Marino et al., 2021). A global study of age-related DNA methylation changes in healthy breast demonstrated that increased methylation primarily occurs at enhancer regions of binding sites for chromatin remodeling genes (Johnson et al., 2017). Epigenetic age of healthy breast is elevated above chronologic age and appears older with other tissues in the body (Horvath, 2013; Sehl et al., 2017), including matched peripheral blood samples from healthy breast tissue donors (Sehl et al., 2017). Estrogen stimulation and chronic cell cycling are thought to drive accelerated aging in breast tissue (Pike et al., 1983; Pike et al., 1993). Risk factors for breast cancer that relate to lifetime estrogen exposure, including earlier menarche and elevated body mass index, are associated with accelerated Grim age in healthy breast tissue (Sehl et al., 2021). Likewise Pan-tissue age, Hannum age, and Phenotypic age in peripheral blood are associated with risk factors for breast cancer, including BMI and alcohol use (Chen et al., 2019). Furthermore, in peripheral blood, an epigenome wide analysis of estimated lifetime estrogen exposure (ELEE) in 216 women in the EPIC-Italy study identified a methylation index of ELEE based on 694 CpGs, and developed a methylation index based on 31 of these most varying CpGs that predicted subsequent breast cancer risk in 440 women with incident breast cancer and 440 controls from the Generations Study (Johansson et al., 2019). An increase of DNA methylationbased ELEE of 1 year was associated with a 5% increase in breast cancer risk (Johansson et al., 2019).

DNA methylation studies comparing normal adjacent breast tissue from women with breast cancer and healthy tissues from cancer-free women revealed epigenetic field effects, with aberrant methylation in specific pathways related to stem cell differentiation, including WNT signaling, known to be epigenetically deregulated in cancer (Teschendorff et al., 2016). Furthermore, epigenetic age in normal adjacent breast tissue from luminal breast cancer patients is increased compared with healthy breast tissue from donors with no history of breast cancer (Hofstatter et al., 2018). In a recent study of 107 breast tumor samples compared with 45 paired adjacent-normal breast tissue samples and 459 normal breast samples, DNA methylation age was estimated using 286 CpGs out of over two million candidate CpGs. Breast tumor samples exhibited age acceleration, appeared 12 and 13 years older than adjacent normal and normal breast tissue with identified pathways involving cellular development and morphology, epidermal growth factor and estrogen receptor signaling (Castle et al., 2020).

Finally, a recent study of epigenetic age-related methylation changes in healthy mammary epithelial tissues demonstrated accelerated epigenetic aging in 12 women with germline mutations in cancer susceptibility genes (Miyano et al., 2021). This study used a breast-specific molecular clock based on methylation of ELF5, a marker critical for mammary development. This finding suggests a link between inherited alterations in DNA repair capacity and accelerated epigenetic aging in tissues at highest risk of developing malignancy.

## Conclusion and Future Directions

DNA methylation-based estimates of biologic age are associated with both cancer risk factors and risk of incident cancer, suggesting a potential mechanistic link between genomic instability, epigenetic age acceleration, and carcinogenesis. Further work is needed to investigate alterations in transcriptomic and proteomic pathways that accompany epigenetic age acceleration prior to the development of cancer. Identification of these changes could lead to targets for chemoprevention in individuals at high risk for cancer. In addition, future studies should identify nonlinear trends in epigenetic age that are associated with cancer risk and modeling epigenetic states that are associated with risk of cancer. Integrative analyses of methylation age along with genomic, transcriptomic, and proteomic data within an individual prior to the development of cancer may ultimately be used to develop predictive tools that could be used to guide risk reduction strategies.
